# The Glycophosphatidylinositol Anchor of the MCMV Evasin, m157, Facilitates Optimal Cell Surface Expression and Ly49 Receptor Recognition

**DOI:** 10.1371/journal.pone.0067295

**Published:** 2013-06-19

**Authors:** Lindsey E. Carlin, Natalya V. Guseva, Michael R. Shey, Zuhair K. Ballas, Jonathan W. Heusel

**Affiliations:** 1 Interdisciplinary Graduate Program in Immunology, University of Iowa, Iowa City, Iowa, United States of America; 2 The Department of Pathology, University of Iowa, Iowa City, Iowa, United States of America; 3 The Department of Internal Medicine, University of Iowa, Iowa City, Iowa, United States of America; 4 The Iowa City VA Medical Center, University of Iowa, Iowa City, Iowa, United States of America; Centre de Recherche Public de la Santé (CRP-Santé), Luxembourg

## Abstract

The murine cytomegalovirus-encoded protein m157 is a cognate ligand for both inhibitory and activating receptors expressed by natural killer cells. Additionally, m157 is expressed on the surface of infected cells by a glycophosphatidylinositol (GPI) anchor. Although endogenous GPI-anchored proteins are known to be ligands for the NK cell receptor, NKG2D, the contribution of the GPI anchor for viral m157 ligand function is unknown. To determine whether the GPI anchor for m157 is dispensable for m157 function, we generated m157 variants expressed as transmembrane fusion proteins and tested cells expressing transmembrane m157 for the capacity to activate cognate Ly49 receptors. We found that the GPI anchor is required for high-level cell surface expression of m157, and that the transmembrane m157 ligand retains the capacity to activate reporter cells and NK cells expressing Ly49H, as well as Ly49I^129^ reporter cells, but with reduced potency. Importantly, target cells expressing the transmembrane form of m157 were killed less efficiently and failed to mediate Ly49H receptor downregulation on fresh NK cells compared to targets expressing GPI-anchored m157. Taken together, these results show that the GPI anchor for m157 facilitates robust cell surface expression, and that NK cells are sensitive to the altered cell surface expression of this potent viral evasin.

## Introduction

Natural killer (NK) cells are innate cytotoxic lymphocytes that participate in the immune responses against a wide variety of microbial pathogens, and also display potent anti-tumor responses [1]–[6]. The activation of NK cells is tightly regulated by an array of activating and inhibitory receptors expressed on their surface, including those of the Ly49 family in rodents (encoded by *Klra* genes) [7]. Inhibitory Ly49 receptors recognize MHC class I and class I-like ligands in both *trans* and *cis*, and control the activation threshold for NK cells at the level of the NK cell immune synapse [8]–[11]. Further, there is promiscuity among the Ly49 receptors for multiple ligands, with a hierarchy of affinities and corresponding inhibitory potency [7], [12], [13]. The activating Ly49 receptors are both fewer in number and, to date, show a reduced ligand complexity compared to the inhibitory Ly49 receptors.

Prominent among the activating Ly49 receptor ligands is the m157 product of murine cytomegalovirus (MCMV), a cell surface glycoprotein attached to the surface of MCMV-infected cells through a glycophosphatidylinositol (GPI) anchor [14], [15]. Like MHC class I, m157 is polymorphic. While m157 expressed by laboratory MCMV strains engages the activating Ly49H receptor of C57BL/6 mice (Ly49H^B6^), forming the molecular basis of the potent *Cmv-1* resistance trait in this mouse strain, it also is a cognate ligand for inhibitory Ly49 receptors, including Ly49I from 129 mice (Ly49I^129^) [14], [16], [17]. In wild strains of MCMV, additional m157 variants have been identified that do not engage Ly49H^B6^ or Ly49I^129^, but are ligands for other inhibitory receptors from various mouse strains, evidence that m157 arose as a decoy ligand, or viral evasin, by exploiting the inhibitory Ly49 receptor-self MHC class I interaction [14], [18], [19]. Notably, as the GPI addition site is highly conserved, all of these m157 variants are predicted to be GPI-associated proteins, raising the possibility that the GPI anchor supports a critical function for m157.

GPI-anchored proteins penetrate only a single layer of the cell membrane, and are usually found in lipid raft microdomains rich in cholesterol and sphingomyelin [20], [21]. In contrast, transmembrane proteins span both layers of the cell membrane and are typically excluded from lipid rafts [22], [23]. While the presence of a GPI anchor for a virally encoded protein is rare, and thus a unique feature for m157, endogenous GPI-associated proteins are well established ligands for other NK cell receptors—most notably NKG2D, an activating lectin-like receptor expressed on all NK cells and a subset of T cells in humans and mice [24]–[27]. NKG2D ligands are a diverse group of cell surface transmembrane and GPI-associated proteins whose expression is normally restricted developmentally, among discrete tissues, or increased in response to viral infection or other cell stresses, increasing cell susceptibility to NK cell cytotoxicity [28]–[30]. Among the retinoic acid early transcript (Raet) gene families of mice (including Rae1α-ε, H60a-c, and MULT-1), and humans (RAET1E, G, and L, and UL16-binding proteins, ULBP1-3), investigators have examined the role of GPI anchors for NKG2D recognition and activation. For example, H60c is GPI-linked, restricted to the skin and shows the lowest affinity for mouse NKG2D among the H60 proteins; yet, H60c has comparable potency to transmembrane H60a and H60b in activating NK cell cytotoxicity when expressed in BaF3-transduced targets [31]. Independent, and more direct examinations of the role of the GPI anchor for the human NKG2D ligands ULBP-1 and ULBP-2 revealed curiously discordant findings. While ULBP-1 may be expressed stably at the cell surface as a transmembrane fusion protein, it is significantly less potent in activating NK cell cytotoxicity, and the authors concluded that NKG2D ligand distribution within the membrane influences the NK cell-target cell interaction [32]. Although ULBP1-3 are normally GPI-anchored proteins, ULBP2 is unique in that a minor fraction exists on the surface as a transmembrane protein. Although the transmembrane ULBP2 isoform shows delayed protein maturation and ultimately is expressed at a lower cell surface density compared to GPI-anchored ULBP2, the potency in activating NKG2D-mediated cytotoxicity is essentially equivalent [33].

The activating Ly49H receptor is a member of the lectin-like NK cell receptor family, but unlike NKG2D, Ly49H has only a single known ligand—MCMV m157. That inhibitory Ly49 receptors recognize endogenous MHC class I ligands, and the observation that m157 and its wild MCMV strain variants engage multiple inhibitory Ly49 receptors, has led to the idea that MCMV uses m157 as a decoy ligand to evade NK cell mediated immunity, and that Ly49H evolved as a means to defeat this viral evasion mechanism [14], [18]. Our previous work has revealed that the Ly49H-m157 interaction is unique in comparison to other related lectin-like receptors and their ligands. Ly49H recognition of m157 is absolutely dependent on m157 residues Ile153 and Lys161; residues that lie outside of the prototypical sites 1 and 2 for inhibitory Ly49 receptor recognition of MHC class I ligands [34]. In addition, N-glycosylation on m157 stabilizes the receptor-ligand interaction, which may be important for prolonged signaling at low ligand densities for m157 on MCMV-infected cells [35]. We wished to extend this analysis by examining the contribution of the GPI anchor to m157 expression and its capacity to functionally engage cognate Ly49 receptors. Herein, we report that MCMV m157 may be expressed as a transmembrane fusion protein, albeit at a reduced cell surface density, and that transmembrane m157 is recognized by both Ly49H^B6^ and Ly49I^129^. However, the potency of transmembrane m157 in activating NK cell cytotoxicity, in particular, is reduced, suggesting that the GPI-anchor of m157 is important for robust surface expression and likely contributes to a stronger and/or prolonged signaling interaction with Ly49H^+^ NK cells.

## Methods

### Mice and Ethics Statement

C57Bl/6 and B6.129S7-*Rag1^tmMom^*/J (*RAG1*-deficient) mice, used as a source for CD4 DNA or fresh splenic NK cells, respectively, were maintained in the University of Iowa Animal Care Unit barrier facility. All mouse experiments were conducted according to protocols approved by the University of Iowa Institutional Animal Care and Use Committee (protocol #0809207). Care was taken to minimize animal suffering.

### Construction of transmembrane m157

RNA was harvested from spleens of C57Bl/6 mice using TRIzol, per manufacturer’s instructions. CD4 cDNA was prepared by RT-PCR using the following primers:

5′- CTCAGAATTCCCAACCAACAAG-3′, 5′-GAGGGAAACCGTGGCCGGTTGTG-3′, 5′-GTCAAGATGGACTCCAGGATC-3′, 5′GAGACTGCGGCCGCATTTATTCTATGAGGGAAACC-3′, 5′-ATAGGATCCGACATGGTCATCGTCCCCCTA-3′, and 5′-CACCCCTCTGGATAAAACGCCACCACGGTTGACATTCCC-3′

**Figure 1:**
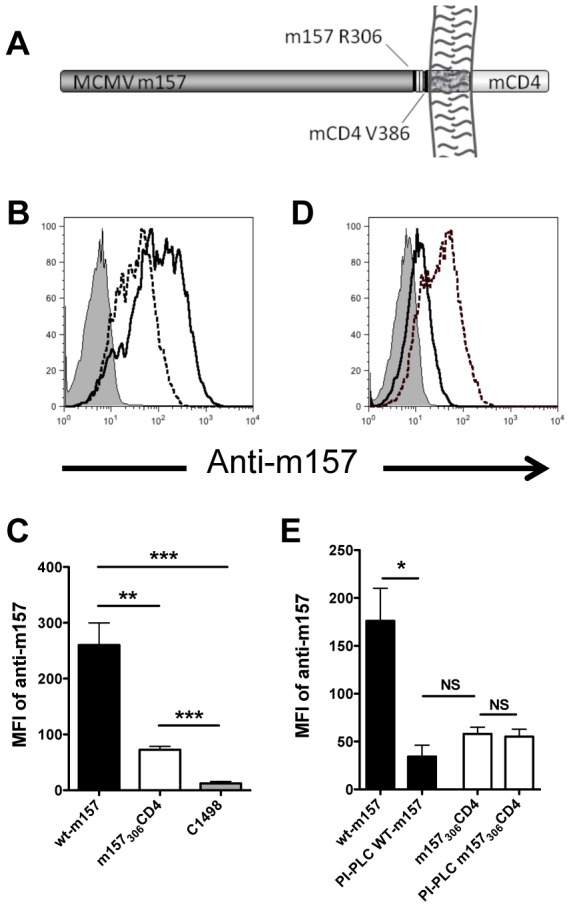
Expression of m157 as a transmembrane protein results in lower surface expression than GPI-anchored m157. C1498 cells were transfected with GPI-anchored m157 (wt-m157) or transmembrane-anchored m157 (m157_306_CD4). (**A**) Schematic of the m157_306_CD4 construct. (**B**) Surface expression of m157 as determined by flow cytometry using the anti-m157 mAb 6D5 in C1498 cells, representative of 7 experiments. Shaded grey histogram shows untransduced C1498 cells, solid line illustrates wt-m157, and dotted line represents m157_306_CD4. (**C**) Mean fluorescent intensity of surface wt-m157 and m157_306_CD4; data are accumulated from 7 experiments. (**D**) C1498 transfectants were treated with PI-PLC and then surface expression of m157 was determined by flow cytometry as in **B**. Data are representative of 3 experiments. (**E**) Mean fluorescent intensity of surface wt-m157 or m157_306_CD4 before and after PI-PLC treatment; data are accumulated from 3 experiments. NS, not significant (*P*>0.05); * *P*<0.05; ** *P*<0.005; *** *P*<0.001 (two-tailed unpaired Student’s t-test). Error bars **C**, **E**, depict standard error of the mean, SEM.

Previous work established the GPI linkage attachment site for m157 is Ser286 [35]. In order to preserve the maximal amount of the m157 extracellular domain, we generated a transmembrane fusion protein with mouse CD4 using PCR-mediated mutagenesis in which the first 306 residues of native m157 are fused in-frame with a diglycine linker to the transmembrane and minimal cytoplasmic domains of mouse CD4 beginning at position Val386 (Fig 1A).

### Cell lines, retroviral transduction, and flow cytometry

The retroviral transduction system including the pMX vectors, retroviral packaging cell line Platinum-E (PLAT-E), BWZ.36 cells expressing an inducible NFAT-lacZ reporter cassette and the derivative reporter line expressing Ly49H, Ly49HI^129^, or Ly49HI^B6^ have been previously described [16], [35]. The Ly49HI^129^ and Ly49HI^B6^ cell lines were a generous gift from Wayne Yokoyama (Washington University, St. Louis, MO, USA). All BWZ.36-derived cell lines were maintained in RPMI 1640 (GIBCO/Invitrogen, Carlsbad, CA, USA) supplemented with 10% FBS (HyClone, Logan, UT, USA), 2 mM L-glutamine, and 10 mM HEPES, NIH-3T3, C1498 (ATCC), and transduced derivatives were maintained in complete DMEM/high glucose (GIBCO/Invitrogen) supplemented with 10% FBS, 1 mM (sodium) pyruvate, 2 mM L-glutamine, and 10 mM HEPES plus 50 µM 2-mercaptoethanol.

Wild-type and mutant m157 cDNA constructs were directionally subcloned into the *Bam*HI and *Not*I sites of the pMX-Puro retroviral vector (pMX vectors and PLAT-E cells kindly provided by Toshio Kitamura, University of Tokyo). Transduced cells were selected in puromycin (2.0 –3.0 µg/ml) for at least 1 wk before use. Cells were surface stained with Cy5-conjugated m157-specific mAb: 6D5, 1F2 [34], or 6H121 [15]. Fluorescently labeled cells were analyzed on a FACSCalibur flow cytometer (BD Biosciences, San Jose, CA, USA) and the data were processed using FlowJo software (Tree Star, Ashland, OR, USA).

### CPRG assay for β-galactosidase activity

Activation of the inducible *lacZ* reporter line, BWZ.36 and the derivative lines HD12 (expressing Ly49H coupled to the DAP12 signaling adaptor), HI129, and HIB6 (a chimeric Ly49H-Ly49I receptor) were assessed as described previously [34], [35]. Conversion of the colorimetric CPRG substrate was quantitatively determined at several points during the linear phase of enzymatic activity by measuring the absorbance at 575 nm (635 nm reference) using a µ-Quant plate reader and K.C.-Jr. software package (Bio-Tek Instruments, Winooski, VT, USA). Results are shown as a percentage of maximal stimulation obtained by culturing each reporter cell line with 5 ng/ml PMA and 1 µM ionomycin.

### Intracellular cytokine staining

B6 RAG1^−/−^ splenocytes were isolated and co-cultured with wt or mutant m157-expressing C1498 or YAC-1 cells in the presence of brefeldin A (10 µg/mL) for 6 hours at indicated ratios in 48-well plates (10^6^ total cells/well) as previously described [35]. Cells were harvested with Versene and surface stained for NK1.1 (mAb PK136) and Ly49H (mAb 3D10, GE Healthcare, Piscataway, NJ, USA). Cells were then fixed (FACS Lysis Buffer, BD), permeabilized, and stained for intracellular IFN-γ (mAb XMG1.2, BD PharMingen, San Diego, CA, USA).

### Chromium release assay

*In vitro* NK cell cytotoxicity was determined by ^51^Cr-release assay as previously described [36]. C1498 and transduced derivatives, and YAC-1 target cells (5×10^3^) were labeled with 10 µCi of ^51^Cr (NEN, Boston, MA, USA), washed, and incubated with B6 RAG1^−/−^ NK cells at indicated effector to target ratios (in triplicate). Cytotoxicity is expressed as the percentage of specific ^51^Cr release: % cytotoxicity  =  [(experimental ^51^Cr release – spontaneous ^51^Cr release)/(maximum ^51^Cr release – spontaneous ^51^Cr release)] x 100. To determine spontaneous and maximum ^51^Cr release, targets were incubated in culture medium alone or in 1% acetic acid. Spontaneous release was always <10% of maximum release. For calculation of lytic units (L.U.), the percentage of specific lysis for each target is plotted against the log_10_ of the E:T ratios. The linear portion of the resulting log_10_ [E:T] vs. %-specific lysis curve is selected and the data entered into a computer program that calculates L.U. using standard linear regression. The E:T ratio required to generate killing of 30% of the targets is obtained from the regression line and used to define a lytic unit of 1.0—the number of NK cell effectors needed to give 30% specific lysis [36].

## Results

### Expression of m157 as a transmembrane protein

To determine the contribution of the GPI anchor of m157 in expression and binding to cognate Ly49 receptors, we created a pMX-PURO retroviral construct that fused the transmembrane and cytoplasmic tail of murine CD4 (beginning at position Val386) to m157 at amino acid Arg306 (m157_306_CD4) (Fig. 1A). Retroviral constructs encoding wt-m157 (GPI anchor) or transmembrane m157_306_CD4 were transduced into C1498 cells and protein expression was verified at the cell surface by flow cytometry using previously characterized anti-m157 antibodies [15], [34]. As shown in Figure 1, the expression of m157_306_CD4 was detectable on the surface of C1498 cells, although the mean fluorescence intensity (MFI) was lower than that of wt-m157 (Fig. 1B, C). To ensure that the difference in protein expression was not restricted to the C1498 cell line, wt-m157 and m157_306_CD4 constructs were expressed in the fibroblast 3T3 cell line. A similar decrease in the surface expression of m157_306_CD4 was observed, suggesting that the decreased cell surface density was intrinsic to the enforced transmembrane expression (Fig. 1B and data not shown).

To confirm that m157_306_CD4 was expressed in the cell membrane as a transmembrane protein, m157_306_CD4 and wt-m157 expressing cells were treated with phosphatidylinositol phospholipase C (PI-PLC), an enzyme that cleaves GPI-anchored proteins from the surface of most cells. C1498 cells expressing wt-m157 or m157_306_CD4 were treated with PI-PLC and then analyzed for m157 expression by flow cytometry (Fig. 1D). While >90% of wt-m157 was cleaved from the surface of C1498 cells (reduced MFI in PI-PLC-treated cells, Fig. 1E), the C1498 cells expressing transmembrane m157_306_CD4 showed no difference in the cell surface anti-m157 MFI (Fig. 1D, E). We observed similar results for additional m157 fusion proteins containing the transmembrane and signaling-deficient cytoplasmic domains from murine DAP12. Analysis of cell culture supernatants for all cell lines expressing transmembrane m157 fusion proteins showed that m157 was not shed from the cell surface, nor was it found to be retained intracellularly (data not shown). Thus, although m157 may be expressed as a transmembrane protein, the level of expression is lower than for wt-m157, suggesting that the GPI anchor of m157 may allow for more efficient or stable expression of the m157 protein at the cell surface.

### Transmembrane m157_306_CD4 activates Ly49H- and Ly49I^129^-expressing reporter cells

**Figure 2:**
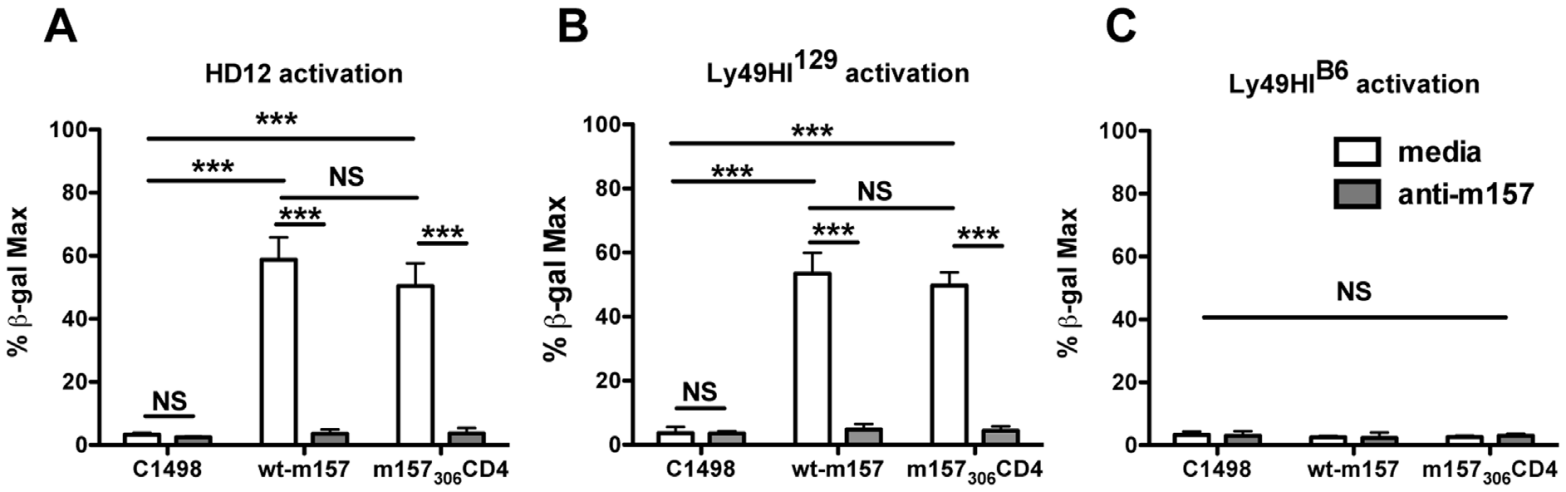
Transmembrane m157 stimulates both activating and inhibitory Ly49 reporter cells. C1498 cells transfected with wt-m157 or m157_306_CD4 were incubated with (**A**) Ly49H^B6^-expressing HD12 cells, (**B**) Ly49HI^129^-expressing cells, or (**C**) Ly49HI^B6^-expressing cells overnight in the presence or absence of anti-m157 mAb (6D5). Results are expressed as the frequency of maximum β-gal production (determined by stimulating cells with PMA and ionomycin). NS, not significant (*P*>0.05); *** *P*<0.001 (two-tailed unpaired Student’s t-test). Results are representative of 3 experiments (error bars depict SEM).

To determine the importance of the m157 GPI anchor in activating the Ly49H receptor, wt-m157 and m157_306_CD4 expressing C1498 cells were incubated with Ly49H^+^ BWZ.36 (HD12) reporter cells. Despite the lower level of surface expression of m157_306_CD4 (Fig. 1B), both m157_306_CD4 and wt-m157 expressing cells were able to stimulate HD12 reporter cells to similar levels (Fig. 2A). This activation of HD12 by either wt-m157 or m157_306_CD4 could be abrogated with the addition of blocking anti-m157 antibodies (Fig. 2A), showing that the activation was m157- specific. Similar results were observed with wt-m157- and m157_306_CD4-expressing 3T3 cells (data not shown). These data indicate that the GPI anchor of m157 is not required for a productive signaling interaction with Ly49H.

Our results show that m157_306_CD4 activates Ly49H-expressing reporter cells, but it was unknown whether the GPI anchor was required for interaction with inhibitory Ly49 receptors, which may engage m157 differently from Ly49H. The extracellular structure of activating and inhibitory Ly49 receptors is similar, as both are composed of homodimeric transmembrane proteins consisting of a stalk region and C-type lectin-like domain (CTLD) [37]. However, it was possible that sequence variations in either the stalk domain or CTLD could result in differential recognition of m157_306_CD4 by activating and inhibitory Ly49 receptors. Therefore, to determine if m157_306_CD4 was likewise capable of interacting with cognate inhibitory Ly49s, a Ly49HI^129^ fusion reporter line was used, in which the CTLD of Ly49I^129^ is fused to the stalk and transmembrane domains of Ly49H^B6^, allowing us to test binding of m157_306_CD4 by the CTLD of an inhibitory NK cell receptor using the same ITAM-reporter system. Both m157_306_CD4 and wt-m157 stimulated the Ly49HI^129^ cell line to similar levels (Fig. 2B). Again, this stimulation was blocked by the addition of anti-m157 (Fig. 2B), demonstrating the m157 specificity of the stimulation. To further demonstrate activation specificity through Ly49I^129^ CTLD binding, m157-expressing cells were incubated with Ly49HI^B6^ reporter cells (a fusion of the Ly49I^B6^ CTLD and the stalk and transmembrane domains of Ly49H^B6^), as Ly49I^B6^ does not bind m157. No activation of Ly49HI^B6^ was observed with either C1498 cells expressing wt-m157, or expressing m157_306_CD4 (Fig. 2C), illustrating that the activation seen with the Ly49HI^129^ reporter cells was not due to interactions between the Ly49H^B6^ stalk domain and m157. These data suggest that the GPI anchor of m157 is not required for a signaling interaction of m157 with Ly49H^B6^ or Ly49I^129^, in which both receptor and ligand are expressed in transduced cells.

### Transmembrane m157 is less potent in stimulating NK cell cytotoxicity compared to GPI-anchored wt-m157 and is associated with impaired Ly49H downregulation

**Figure 3:**
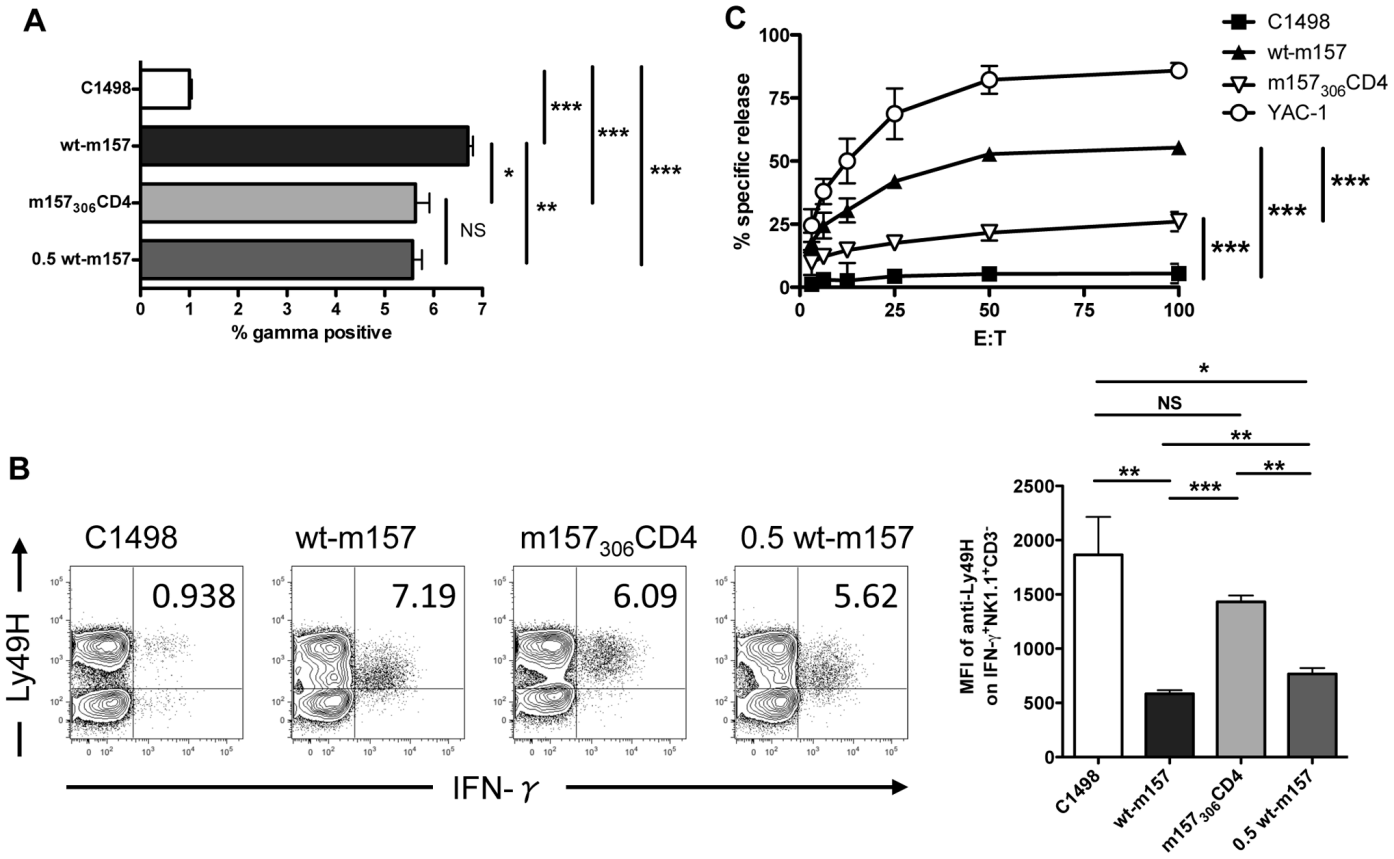
Transmembrane m157 expression impairs NK cell Ly49H receptor downregulation and cytotoxicity, but not IFN-γ production. C1498 transfectants were incubated with B6 RAG1^−/−^ splenocytes. (A) IFN-γ production of NK1.1^+^CD3^−^Ly49H^+^ gated cells was determined by flow cytometry. (B) Representative plots showing downregulation of Ly49H, and mean fluorescent intensity of Ly49H on NK1.1^+^CD3^−^IFN-γ^+^ cells (bar graph). Numbers in upper right quadrant are equal to the frequency of IFN-γ^+^ cells of Ly49H^+^ NK cells. NS, not significant (*P*>0.05); * *P*<0.05; ** *P*<0.005;*** *P*<0.001 (two-tailed unpaired Student’s t-test). (C) NK cell cytotoxicity against m157 transfectants was determined by chromium release assay. B6 RAG1^−/−^ splenocytes were incubated with Cr^51^-labeled targets at indicated ratios. *** *P*<0.001 (two-way ANOVA with Bonferroni post-tests). Data are representative of at least two independent experiments (error bars A, B, depict SEM).

While our results demonstrate that m157_306_CD4 interacts with Ly49H^B6^ and can result in reporter cell activation, we wanted to assess the role of the m157 GPI anchor in detection of m157 by Ly49H-expressing NK cells. To determine whether the GPI anchor of wt-m157 is similarly dispensable for NK cell activation, we incubated wt-m157 or m157_306_CD4-expressing C1498 cells with B6 RAG1^−/−^ splenocytes (lacking B cells and T cells) and measured activation of NK cells by intracellular IFN-γ production. A significant decrease in the frequency of IFN-γ ˜-producing Ly49H^+^ NK cells was observed when stimulated with m157_306_CD4-expressing C1498 cells (Fig. 3A). In addition, a striking difference in apparent Ly49H receptor downregulation was observed for splenocytes incubated with wt-m157 targets vs. those expressing transmembrane m157_306_CD4: wt-m157 stimulated a rapid and robust downregulation of cell surface Ly49H in the responding NK cells, whereas transmembrane m157 showed little capacity to downregulate Ly49H (Fig. 3B).

Next, to determine whether the GPI anchor of m157 contributes toward a cytotoxic response by Ly49H^+^ NK cells, killing of wt-m157- or m157_306_CD4-expressing C1498 cell targets by B6 RAG1^−/−^ splenocytes was measured using a traditional chromium-release assay. Cytotoxicity stimulated by the transmembrane m157_306_CD4-expressing cells was higher than background (parental C1498 targets), but it was significantly lower than the cytotoxicity stimulated by wt-m157-expressing targets (Fig. 3C). While these NK cell results suggest that the GPI anchor of m157 is important in inducing a strong activation signal for NK cell effector functions, these observations may also be related to the reduced cell surface density of transmembrane m157_306_CD4 expression on the surface of C1498 cells (Fig. 1B and 1C). Therefore, to control for the decreased cell surface density if m157_306_CD4, the ratio of wt-m157 expressing C1498 cells to B6 RAG1^−/−^ splenocytes was adjusted so that similar amounts of surface m157_306_CD4 and wt-m157 (0.5 wt-m157) were present in the reaction wells. The frequency of IFN-γ-producing Ly49H^+^ NK cells stimulated by a reduced number of wt-m157 expressing C1498s was now similar to that of m157_306_CD4-stimulated NK cells (Fig. 3A, 3B). However, the reduced number of wt-m157 targets does not normalize Ly49H receptor downregulation (Fig 3B); wt-m157 and 0.5 wt-m157 targets drive comparable Ly49H downregulation and are significantly different from that observed with the transmembrane m157_306_CD4-expressing targets (Fig 3B, bar graph representation). This difference in activation of NK cell effector function mediated by GPI-anchored vs. transmembrane m157 is also evident in the cytotoxicity experiment (Fig 3C). For example, the increase in percent-specific ^51^Cr-release for wt-m157 targets at E∶T of 12.5∶1 to 25∶1 (11.5±1.8) and from 25∶1 to 50∶1 (10.8 ±1.2) is significantly greater than that observed for targets expressing the transmembrane m157 at the same E:T points (12.5-to-25  =  2.7±0.3 and 25-to-50  =  4.0 ±1.0, p≤0.005, student’s t-test). To more rigorously compare the efficiency of NK cell killing between the GPI-anchored and transmembrane m157 targets, we calculated lytic units (L.U.) based upon the lytic activity required to kill 30% of the targets, as previously described [36]. The splenic NK cells display 25-fold greater killing activity against C1498 targets expressing GPI-anchored m157 (20.8 L.U.) compared to the same cells expressing transmembrane m157 (0.7 L.U.; activity vs. YAC controls  =  50.3 L.U.). This decreased cytotoxity against the transmembrane m157 targets is much greater than expected based solely upon a difference in surface m157 density.

## Discussion

Although several endogenous MHC class I-like ligands for NKG2D are known to be GPI-linked, the GPI anchor of m157 is uncommon; the only other viral protein reported to be expressed on an infected cell with a GPI anchor is NS1 of dengue virus [38]. This rare feature of m157 prompted us to test whether the GPI anchor was necessary for expression, or required for productive interactions with cognate Ly49H^B6^ or Ly49I^129^ receptors.

In the absence of other MCMV proteins, the GPI anchor of m157 is not strictly required for expression in transduced cell lines, as we were able to detect surface expression of m157_306_CD4 in both C1498 and 3T3 cell lines (Fig. 1B and data not shown). In addition, we expressed other versions of transmembrane m157 fusion proteins where m157 was fused to a transmembrane partner at the upstream omega site (17 amino acids upstream from Ser307), and were able to detect cell surface expression, albeit transiently (data not shown). These experiments indicated that the entire extracellular domain of m157 (through R306) was necessary for stable, long-term cell surface expression. However, the surface expression of m157_306_CD4 was significantly lower than that of wt-m157 (MFI  =  260 ± 88 vs. 72 ± 13). Interestingly, m157 expression on MCMV-infected cells is far lower than that of wt-m157 in our transduced cell lines [15], suggesting that the cell surface expression of m157 is very tightly regulated in the context of MCMV viral infection. Thus, during MCMV infection, the function of the GPI anchor in facilitating m157 expression potentially could be important in maintaining a critical threshold density for optimal Ly49 receptor binding. To test this hypothesis, we attempted to generate recombinant MCMV strains expressing the transmembrane m157_306_CD4 variant. Unfortunately, recombinant viruses with the desired m157 mutation were not isolated.

Detection of m157 by Ly49H^+^ NK cells is critical for MCMV-resistance of C57Bl/6 mice, and deletion of either m157 or Ly49H results in lethal (BALB/c-like) susceptibility due to decreased control of MCMV replication [39], [40]. Although IFN-γ has been shown to be important in control of MCMV virus infection at early time points [41], Ly49H-deficient NK cells are equally capable of producing IFN-γ as wt NK cells within the initial 38 hours of MCMV infection, suggesting that m157 recognition by Ly49H^+^ NK cells is not required for IFN-γ production early in infection [39], [42]. Although our observation that the reduced MFI of transmembrane m157_306_CD4 is associated with a reduced stimulation of IFN-γ by NK cells, we believe it is unlikely that the transmembrane anchor of m157 would alter IFN-γ production during MCMV infection. Rather, Ly49H^+^ NK cell-mediated protection from MCMV infection in C57Bl/6 mice is dependent on perforin [41]. Consistent with this, we observed decreased efficiency of killing of m157_306_CD4-bearing target cells by freshly isolated B6 RAG1^−/−^ splenocytes, possibly reflecting a decreased strength and/or duration of the Ly49H-m157 interaction when the m157 GPI anchor is replaced by a transmembrane domain.

Our results also demonstrate that the transmembrane form of m157 lacks the capacity to downregulate Ly49H on NK cells, which occurs readily when using transduced cells expressing wt-m157 (Fig. 3B), and is dependent, in part, upon N-glycosylation of m157 [35]. Here, impaired downregulation of the Ly49H may have been affected by a lower transmembrane m157 density, as supported by experiments in which the number of productive interactions between wt-m157 and Ly49H are gradually reduced by titration of a blocking anti-m157 antibody (data not shown). However, we observed little or no Ly49H receptor downregulation in our experiments with the m157_306_CD4-expressing cells, which suggests that even a reduced number of m157-Ly49H interactions are qualitatively lacking. Further, since cell surface receptor downregulation is often attributable to receptor endocytosis, the GPI anchor of m157 may regulate the m157-Ly49 receptor interaction both by affecting the number of available cell surface interactions and the sequestration of Ly49 receptors with potential signaling adaptors or downstream signaling factors within endocytosed compartments [43], [44]. Indeed, endocytosis of activated receptors can enhance activation of ERK1/2 [43], a signal component required for NK cell cytotoxicity [45], and activated by the Ly49H adaptor molecule, DAP12 [46].

Our experiments using the chimeric Ly49I^129^H-expressing reporter cells suggest that the GPI anchor of m157 is not required for productive binding between m157 and an inhibitory Ly49 receptor. Our previous work has highlighted the unique binding characteristics of the Ly49H-m157 interaction [34], [35], but less is known about how m157 is interacting with its multiple inhibitory Ly49 receptor counterparts. For these interactions, drift in the CTLD residues for inhibitory Ly49 receptors is matched by substitutions in the extracellular domain of m157 [18], but precise binding sites have not been identified. The functional consequences for inhibitory Ly49 receptors engagement of transmembrane m157 are more difficult to assess, and are likely to be confounded by varying affinities between inhibitory Ly49 receptors and their ligands.

As has been reported for GPI-anchored ligands for NKG2D, expression of m157 as a transmembrane protein results in a lower cell surface density of the ligand. We were unable to demonstrate accumulation of intracellular m157, or enhanced secretion of m157 into the culture medium of m157_306_CD4-transduced transduced cells (data not shown). Thus, it is our interpretation that the GPI anchor of m157 contributes to the translation efficiency and/or overall stability of the protein. The post-translational processing, trafficking, and turnover of GPI-associated proteins are complex, and likely operate differently among cell types (e.g., epithelial vs. hematopoietic cells infected with MCMV) [47], [48]. Regardless, the reduced surface density and potentially altered membrane distribution for transmembrane m157 may provide a synergistic effect to decrease the robustness of the Ly49H-m157 interaction, involving the number of clustered events, the duration of Ly49H-m157 binding, or both.

In summary, we demonstrate that the GPI anchor for MCMV m157 contributes to the cell surface expression and the potency of its functional interaction with Ly49H^+^ NK cells. These results are consistent with the emerging model for NK cell activation in which activation of potent effector functions is highly regulated by a diverse array of inhibitory and activating receptors, resulting in rheostatic, or tunable, signal integration that must function at the level of the NK cell immune synapse—an idea that is extremely difficult to test, but has been simulated *in silico* [49], [50]. The individual contribution of a given receptor-ligand interaction itself is tuned by factors such as inherent affinity, glycosylation, density and membrane distribution. It is at this level where the role of GPI-anchoring is most likely affecting the NK cell immune response.
